# Flexible thermal interface based on self-assembled boron arsenide for high-performance thermal management

**DOI:** 10.1038/s41467-021-21531-7

**Published:** 2021-02-24

**Authors:** Ying Cui, Zihao Qin, Huan Wu, Man Li, Yongjie Hu

**Affiliations:** Department of Mechanical and Aerospace Engineering, University of California, Los Angeles (UCLA), Los Angeles, CA 90095 USA

**Keywords:** Energy, Materials for energy and catalysis

## Abstract

Thermal management is the most critical technology challenge for modern electronics. Recent key materials innovation focuses on developing advanced thermal interface of electronic packaging for achieving efficient heat dissipation. Here, for the first time we report a record-high performance thermal interface beyond the current state of the art, based on self-assembled manufacturing of cubic boron arsenide (s-BAs). The s-BAs exhibits highly desirable characteristics of high thermal conductivity up to 21 W/m·K and excellent elastic compliance similar to that of soft biological tissues down to 100 kPa through the rational design of BAs microcrystals in polymer composite. In addition, the s-BAs demonstrates high flexibility and preserves the high conductivity over at least 500 bending cycles, opening up new application opportunities for flexible thermal cooling. Moreover, we demonstrated device integration with power LEDs and measured a superior cooling performance of s-BAs beyond the current state of the art, by up to 45 °C reduction in the hot spot temperature. Together, this study demonstrates scalable manufacturing of a new generation of energy-efficient and flexible thermal interface that holds great promise for advanced thermal management of future integrated circuits and emerging applications such as wearable electronics and soft robotics.

## Abstract (web summary)

Well-developed prototype interface materials for electronics thermal management are limited to a low thermal conductivity or high elastic modulus. Here, the authors report flexible thermal interfaces through self-assembled manufacturing of polymetric composites based on the high thermal conductivity of cubic boron arsenide.

## Introduction

**Fig. 1:**
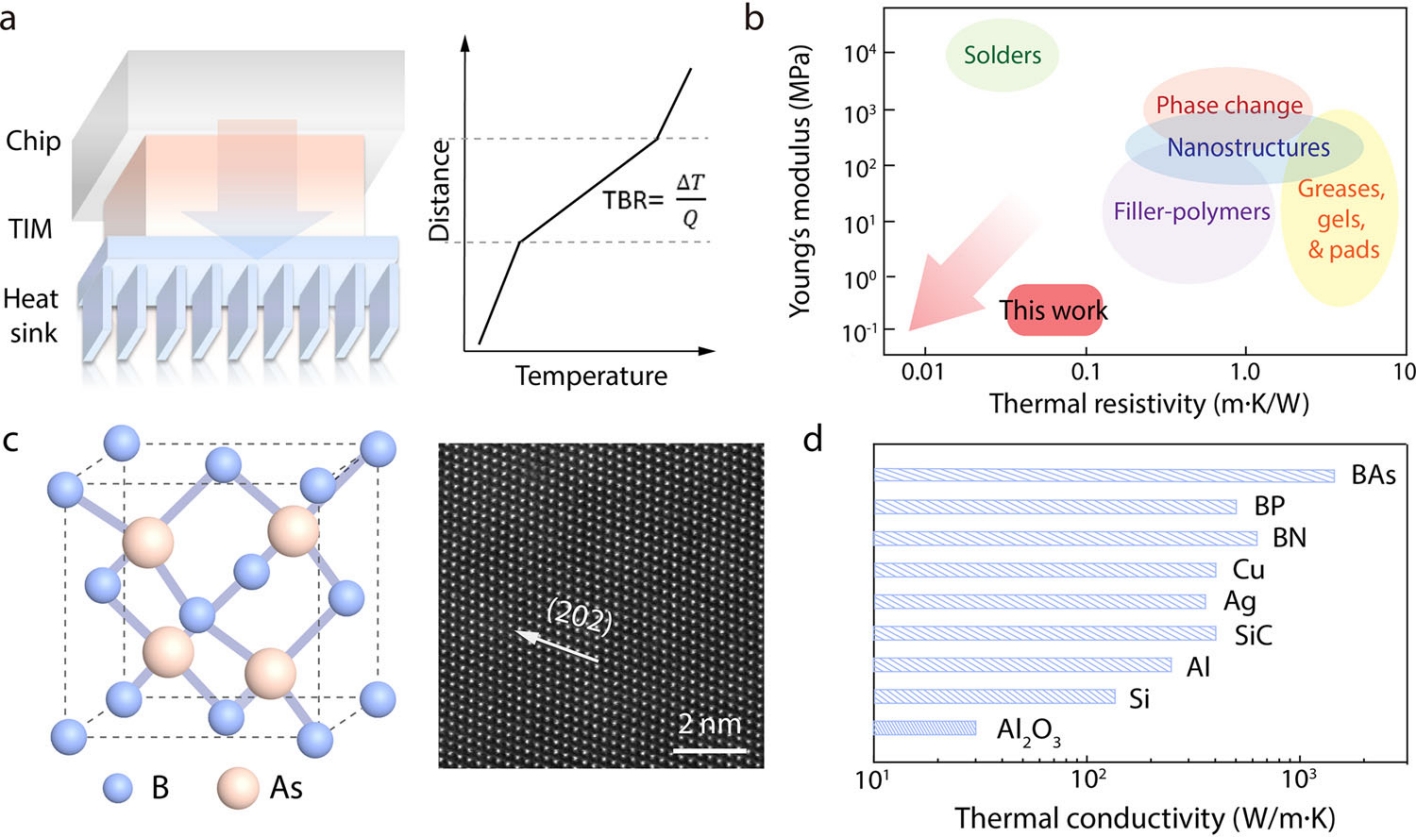
High-performance thermal interface based on self-assembled boron arsenide (s-BAs) to enhance heat dissipation. **a** Schematic illustration of a typical thermal interface applied in electronic packaging. Heat dissipation from the chip to heat sink via the thermal interface, unusually limited by the resulting thermal boundary resistance (TBR)^33^. ΔT is the temperature drop and Q is the heat flux across the interface. **b** Performance comparison of s-BAs vs. the state-of-the-art. Arrow pointing to the bottom left indicates the design goal of high-performance thermal interfaces to achieve both low elastic modulus and low thermal resistivity (i.e., high κ). **c** Schematic of the zinc-blende crystal structure of cubic BAs and its high-resolution TEM image showing atomically resolved lattices. The arrow indicates the crystal direction of (202). **d** Thermal conductivity distribution of different materials, including typical fillers.

Heat dissipation has been a critical technology challenge for modern electronics for decades^1–7^. With information technology ramping up in an increasingly digitalized world, electronics cooling is scaling up rapidly in its impact on global energy consumption^8,9^. For instance, current data centers consumes over 200 TWh (terawatt-hour) of electricity annually but more than 50% of the total electricity is used for cooling, i.e., removing waste heat rather than for data storage or computing^10,11^. In all hierarchical electronic systems ranging from nanoscale transistors, smart phones, laptops, vehicle electronics, to data server farms, waste heat dissipates from the hot spots to heat sink across a series of thermal resistance of multiple device layers and their interfaces. As a result, the device performance, reliability, and energy efficiency can be strongly degraded by a large thermal resistance and a rising hot spot temperature. To address this challenge, recent key research focus for thermal management aims to develop thermal interfaces that enhances thermal coupling and minimize thermal resistance between heterogeneous components^10^. In general, high-performance thermal interface requires both high thermal conductivity (κ) and low elastic modulus (*E*). When inserted between an electronics layer and a heat sink (Fig. 1a), high κ minimizes thermal resistance and enhance heat dissipation, and low *E* enables good surface compliance, thermal contact area, and thermomechanical stability. Current commercial thermal interfaces, however, are usually limited by low κ ~ 1 W/m ∙ K or high *E* ~ 1 GPa, which largely constrains the cooling performance. In addition, emerging applications like wearable electronics and soft robotics require their thermal interfaces to be soft and flexible, but has yet to be explored^12–14^.

## Results and discussion

During the last decades, intensive research efforts have been devoted in the area and progress has been exemplified by varied categories including thermal greases, gels, pads, tapes, conductive adhesives, phase change materials, metallic solders, etc., with the understanding that different thermal interface materials may have their unique applications. The state-of-the-art performances of thermal interfaces are summarized in Fig. 1b. Fundamentally, there is trade-off between high thermal conductivity and soft mechanics^15,16^. Strongly bonded materials such as ceramics and dielectrics usually give high κ^17^, however, their rigid structures can potentially lead to performance degradation like mechanical pump-out, delamination, cracking, and void formation. On the other hand, soft materials such as polymers can provide effective interface contact but are usually limited by an intrinsically low κ ~ 0.2 W/m ∙ K^18,19^. For example, metal solders (e.g., indium)-based interfaces provide good thermal conductivity but their applications are largely limited due to high *E* ~ 1 GPa or above; in addition, solder systems are usually not applicable when electrical insulation is required. Nanostructures such as carbon nanotubes and metal nanowires have been applied to make compromise and improve the mechanical compliance^20–23^. Adhesives and gels possess good mechanical compliance, but usually exhibit a low thermal conductivity; their mixtures have been studied to make improvement over poor interfaces and weak van der Waals bonding^16,24–26^. Despite many exciting progresses (Fig. 1b), high-performance thermal interfaces with the combination of low elastic modulus, large flexibility, and high thermal conductivity have remained to be demonstrated^16^. In the meanwhile, thermal management has been calling on the development of new materials with ultrahigh thermal conductivity^27^. Recently, building on ab initio theoretical calculations^28–31^, a new class of boron compound semiconductors^3–7,32^, including boron arsenide (BAs) and boron phosphide (BP), has been discovered with ultrahigh thermal conductivity beyond most known heat conductors (Fig. 1c, d). In particular, cubic BAs has a record thermal conductivity over three times that of the industrial high conductivity standards such as copper and SiC, and twice higher than cubic boron nitride^3,32^. With the great application promise in thermal management, the development of BAs for thermal interface, however, has not been explored due to its recent discovery. Here, we report highly flexible thermal interfaces through self-assembly based manufacturing of polymetric composites by taking advantage of the ultrahigh thermal conductivity of BAs crystals. As demonstrated through thermal and mechanical characterizations, the BAs thermal interface exhibits record-high performance with an unprecedented combination of high thermal conductivity (κ ~ 21 W/m ∙ K), excellent elastic compliance similar to that of soft biological tissues (*E* ~ 100 kPa), and high flexibility that are beyond the current state-of-the-art and could lead to advanced thermal management of solid-state and flexible electronics.

**Fig. 2:**
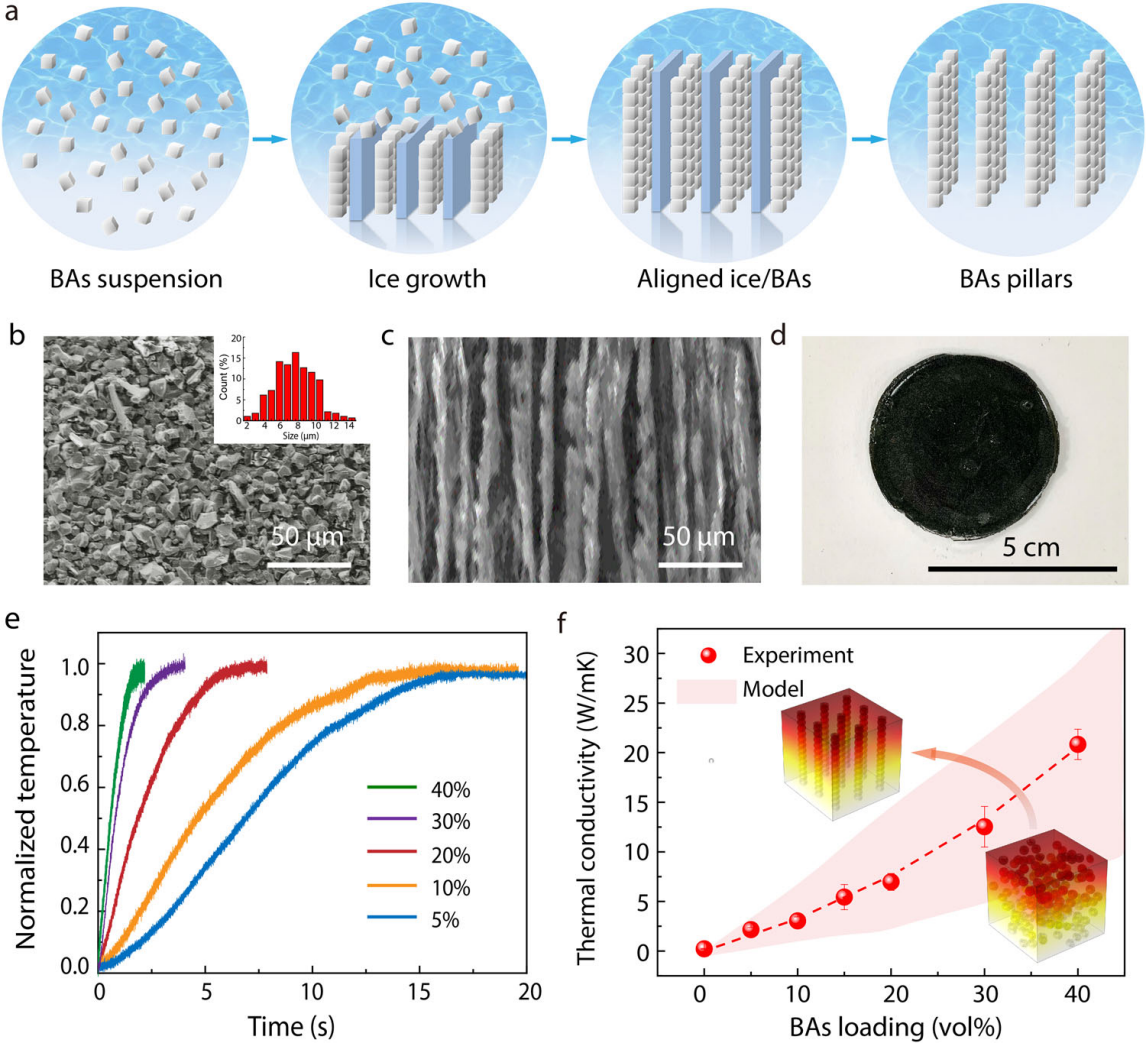
Self-assembly based manufacturing and thermal measurement of s-BAs. **a** Schematic illustrating the self-assembly process through freeze-drying of BAs suspensions to form aligned BAs pillars and polymetric composites. **b** SEM images of as-synthesized BAs crystals. Inset indicates the crystal size distribution. **c** Cross-section SEM image of the s-BAs, verifying an aligned lamellar structure. **d** Optical image of an inch-size s-BAs sample. **e** Laser flash measurement of the s-BAs samples with different BAs loading ratios. **f** Thermal conductivity of s-BAs with different BAs loadings. The red symbols are experimental data, and the pink shadowed background represents the modeling results considering varied extents of alignment.

To achieve high performance, we first carefully examine the structural design of BAs particles to achieve efficient heat-dissipation pathways. Based on literature experience^16^, it has been shown that structural optimization is critical to the thermal conductivity of thermal interfaces: Polymer matrixes are generally soft to enable mechanical compliance, but their intrinsic low thermal conductivity (~0.2 W/m ∙ K) could reduce the overall thermal conductivity. In particular, when high conductivity fillers are randomly distributed, the heat-transfer paths in polymer could be significantly elongated and thereby minimize the contribution from fillers^16^. In addition, the organic–inorganic interfaces could result in thermal boundary resistance due to mismatch in phonon spectra and density of states between heterogeneous components^33–35^. As a matter of fact, this explains why typical industrial thermal interfaces have a low conductivity around 1 W/m ∙ K or below. To quantitatively evaluate the effect from structural design on the overall thermal conductivity, we performed multiscale simulation to calculate the thermal conductivity of the composite materials with varying extents of alignment of BAs fillers (Fig. 2f)^33^. The alignment is quantified by the standard deviation of distance (σ) from the BAs particles to the centerline of the alignment pillar, with σ approaching 0 for perfect alignment and increased σ for disorders^36^. A temperature gradient is applied across the structure to compute the volume-averaged heat flux density over the whole domain using the finite element analysis (Methods and Supplementary Information). The effective thermal conductivities of self-assembled boron arsenide (s-BAs) with varied extents of alignment are determined and plotted in Fig. 2f (pink shadowed background). The thermal conductivity and specific heat used in this simulation are all measured from experiment. The simulation results indicate that an effective design to achieve aligned fillers could effectively enhance the overall thermal conductivity of s-BAs.

To achieve rational alignment of BAs structures in the thermal interface, we designed a self-assembly based manufacturing method using the ice-template process (Fig. 2a). First, BAs particles were dispersed to form an aqueous suspension. The BAs aqueous slurry was subsequently transferred into a tube mold. A directional temperature gradient (e.g., from dry ice bath at the cold side to room temperature at the high temperature side) was applied across the mold that led the slurry to freeze. At the cold side, the nucleation of the ice crystals started and led to the formation of a constitutionally super-cooled zone directly ahead of the growing ice front. Such an unstable region eventually resulted in perturbations, breaking the planar ice front into a columnar structure; consequently, ice crystals start to follow the temperature gradient direction to gradually grow into aligned lamellar pillars. In the meanwhile, as driven by the growth of ice template^37,38^, BAs crystals are expelled and enforced into assembled arrays to replicate the morphology and fill the interspacing between the ice pillars. After this self-assembly process is complete, we dehydrated the sample using a freeze-drying process so that the aligned BAs structures were maintained. Thermodynamically, the freeze-drying process uses a low pressure and a low temperature below the equilibrium triple points in the phase diagram of water (i.e., 273.16 K and a partial vapor pressure of 611.657 Pa) to achieve sublimation of solid ice directly to vapor without going through a liquid phase, so that the structural distortion is minimized^37–39^. Finally, polymer melt was infiltrated into the BAs assembly and solidifies to enhance the mechanical supporting and form composite s-BAs. The resulting structures were carefully verified by cross-section scanning electron microscopy (SEM) images before (Fig. 2b) and after the assembly process (Fig. 2c), indicating that the aligned lamellar network of BAs pillars can be formed and well maintained during the processing. Note that this manufacturing approach allows readily preparation of inch-size s-BAs samples and further scaling up (Fig. 2d).

The thermal conductivity of s-BAs was measured using the standard laser flash method^40^. Figure 2e shows typical temperature rise curves for s-BAs with varied BAs volumetric loadings of 5%, 10%, 20%, 30%, and 40%, respectively. We have included detailed measurement results in Supplementary Table S1. The measurement verifies the high thermal conductivity of s-BAs (Fig. 2f): For example, a record-high thermal conductivity of 21 W/m·K has been measured for 40 vol% s-BAs, which represents ~20 times enhancement over typical thermal epoxies and greases as current industrial thermal interface standard. Consistent with our modeling design, the experimental results also show that the thermal conductivity of s-BAs was enhanced by over 400% through the self-assembled alignment versus random distribution. We also found that the overall thermal conductivity of the assembled s-BAs is dominated by that of BAs fillers, regardless of typical polymer matrix (elastomer, epoxy, etc.). Taking a typical thermal interface thickness of 100 μm, this leads to a total thermal resistance of 0.05 K·cm^2^/W, which is below most of the literature reports. For example, traditional materials based on greases, adhesive, gels, and phase change materials typically yield a higher resistance in the ranges of about 0.2–1, 0.15–1, 0.4–0.8, and 0.3–0.7 K·cm^2^/W, respectively^24^. Elastomic pad, silicone sheet, and thermal tapes typically have the total resistance range of about 1–4 K·cm^2^/W. The demonstrated performance of s-BAs is also record-high among other composites with various fillers including metals, ceramics, semiconductors, oxides, and nanomaterials^16^.

**Fig. 3:**
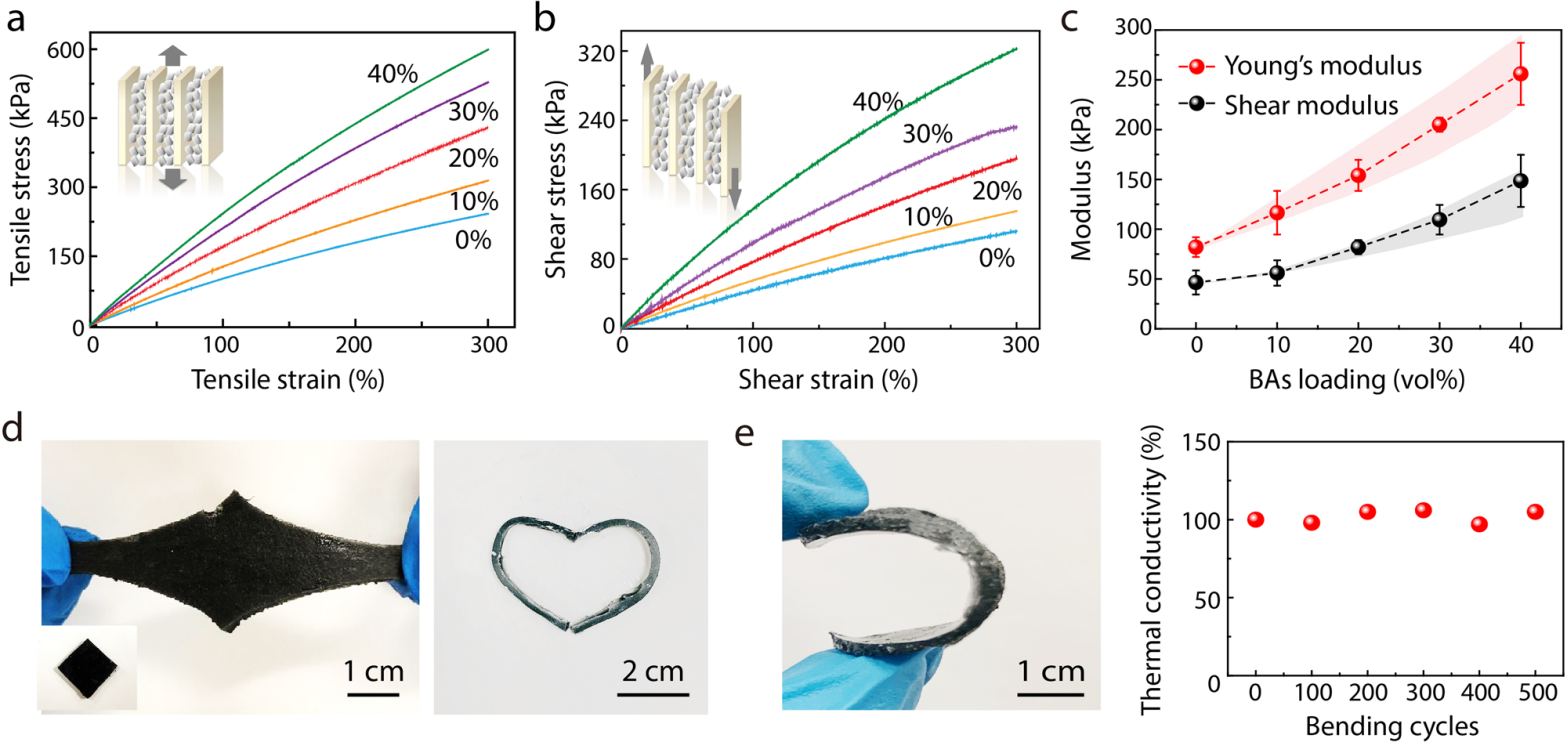
Mechanical measurements and high flexibility of s-BAs. Representative stress–strain experimental curves for: **a** Young’s modulus and **b** shear modulus measurements, with varied loading ratios. **c** The Young’s modulus and shear modulus of s-BAs. The solid symbols are experimental data, and the shadowed backgrounds represent the modeling results considering varied extents of alignment. **d** Optical images of the highly flexible s-BAs. Inset on the bottom left, indicates the original size. **e** Bending tests and thermal conductivity measurement of s-BAs in response to the cyclic bending.

In addition to high thermal conductivity, high mechanical compliance is another critical property for high-performance thermal interface. The capability of deformability between interfaces leads to the most fundamental engineering requirements, i.e., low elastic modulus to allow shape change and conformal interfacial contact. In addition, concerning the practical application in electronic packaging, low Young’s modulus supports flexible functionality of thermal interfaces in different directions. We performed the Young’s modulus and shear modulus measurements of the s-BAs samples with varied BAs loading ratios from 0% to 40% (Methods). The shear modulus was assessed by the lap-shear adhesion test. The representative stress–strain curves from the measurements are shown in Fig. 3a, b. The Young’s modulus and shear modulus are determined by the slope of the loading curve at a nominal strain of 5% and plotted in Fig. 3c. These measurement results verify that the s-BAs remains soft with BAs loading volumes up to 40 vol%, with the shear modulus slightly increased from 47 to 148 kPa, and the Young’s modulus from 82 to 256 kPa. The s-BAs can support uniaxial strains above 500%, similar to that of a homogeneous elastomer. These results indicate that the overall BAs/elastomer composite still remains good mechanical compliance. The mechanical properties are also evaluated using the finite element method by treating the s-BAs as a composite with the experimental structures. The Young’s and shear modulus are determined by computing the structural deformations under applied force along the axial and shear directions, respectively (Methods). As shown in Fig. 3c, there is a good agreement between the simulations (shadowed backgrounds) and experiments (solid symbols), indicating that the BAs particles are uniformly distributed in the composite.

Further, we demonstrate the high flexibility of the s-BAs. A highly flexible thermal interface with both high thermal conductivity and high flexibility is required for thermal management applications in flexible electronics, soft robotics, and other emerging areas, which however remains to be demonstrated^12–14^. As shown in Fig. 3d, the s-BAs can be highly deformable to support uniaxial strains more than 500% stretching over its original size. In addition, the s-BAs can be compressed to random geometries such as a heart-shape circle (right, Fig. 3d) without leading to a mechanical breakdown, which is impossible for standard thermal interface materials. To further explore the potential application in flexible devices, we have performed thermal measurement of the s-BAs under cyclic mechanical bending of the sample (Fig. 3e), verifying the preserved high thermal conductivity. The thermal conductivity of our s-BAs sample maintains stability over at least 500 bending cycles with a maximum fluctuation within 7%. The persistent high thermal conductivity actually indicates the robust structures during bending tests, as verified by the cross-section SEM images taken after bending cycles (Supplementary Figure 1). The retention of highly efficient heat dissipation after mechanical bending underscores the promise of using s-BAs for thermal management of flexible devices.

**Fig. 4:**
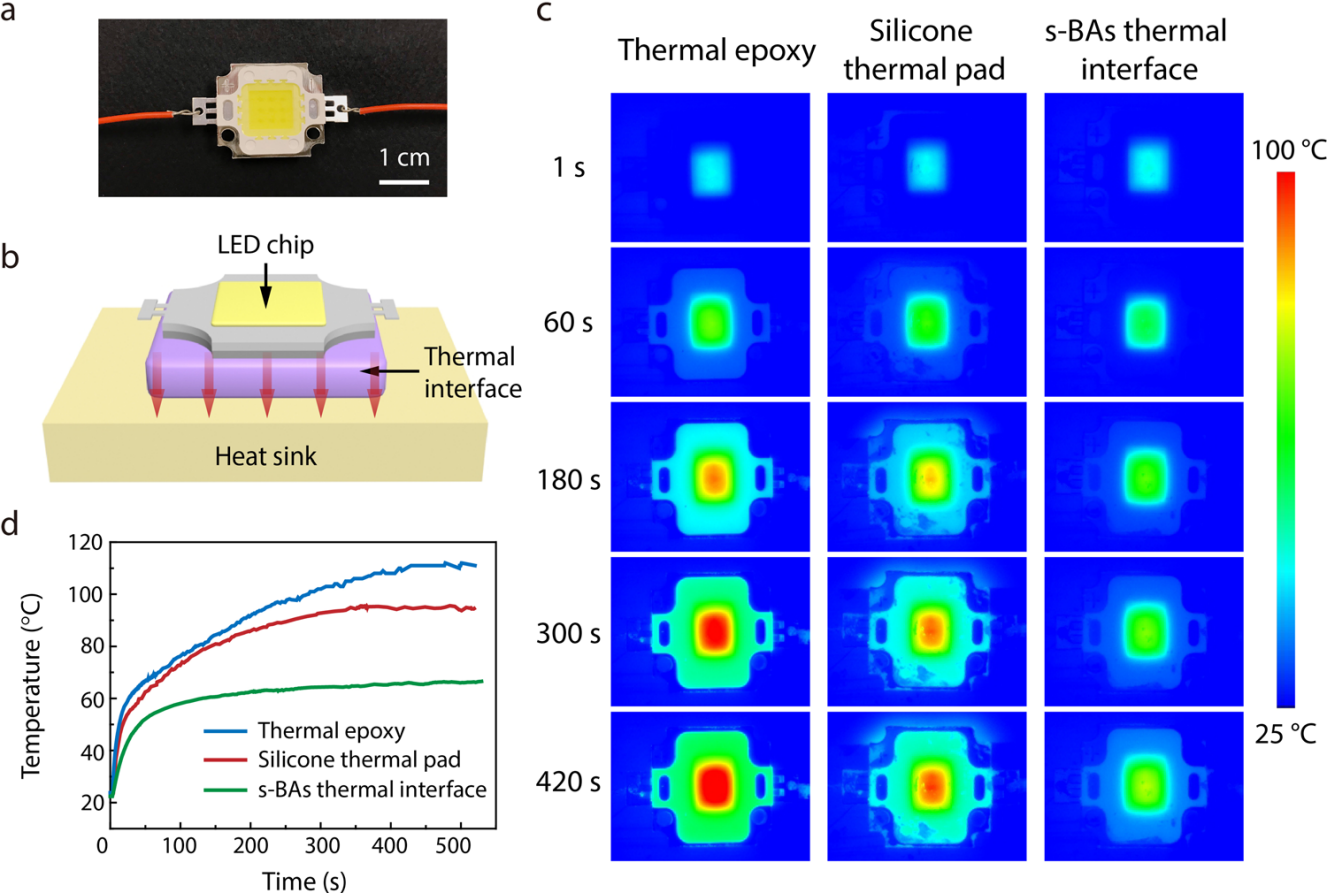
Device demonstration of using s-BAs for high-performance thermal management. **a** Optical image of a light emitting diode (LED) and **b** schematic illustration of its integration with a thermal interface and heat sink. **c** Time-dependent infrared images of the LED integrated with different materials (thermal epoxy, silicone thermal pad, and s-BAs), indicating temperature distributions near the hot spot. **d** Comparison of the LED hot spot temperatures using different thermal interface materials.

As a further step, we demonstrated a proof of concept experiment to verify the superior device-cooling performance of the s-BAs, through the integration and in situ characterizations of a LED during its operation (Fig. 4). To make direct comparison, three types of thermal interfaces, i.e., the commercial thermal epoxy, silicone sheet, and our s-BAs (Methods) are integrated as sandwiched between a 10 W LED chip and a copper heat sink (Fig. 4a, b). Note that all thermal interfaces were chosen to be with the comparable size, thickness, etc. settings. An infrared camera was used to record the surface temperature of the LED chips, with the Cu heat sink maintained at the room temperature (23 °C). Figure 4c shows a series of infrared images after lighting up the LED chips, measuring the transient temperature dependence. With thermal epoxy and commercial silicone sheet, the chip surface temperature increased up to ~110 and 95 °C, respectively. In contrast, the stable temperature is much lower (~65 °C) when the BAs composite was used as the thermal interface. Quantitatively, the time-dependent surface temperature of the LED chip was measured based on the infrared images and plotted in Fig. 4d, showing a dramatic increase for the devices integrated with thermal epoxy and silicone sheet comparing to that with s-BAs. The large contrast in hot spot temperature difference clearly demonstrates the superior cooling capability of the developed s-BAs for future thermal management applications.

In conclusion, we developed a high-performance thermal interface material fabricated through a scalable self-assembly based manufacturing of the recently developed high-thermal-conductivity BAs for advanced thermal management. The s-BAs exhibits an unprecedented combination of high thermal conductivity (21 W/m · K) and an excellent elastic compliance similar to that of soft biological tissues (elastic modulus ~100 kPa). Our thermal and mechanical experiments together with multiphysics modeling show that, upon the designed alignment of BAs crystals, the thermal interface preserves efficient heat-transfer paths while maintaining the high mechanical compliance of polymer matrix. Moreover, the s-BAs shows high flexibility that could be applied to emerging applications such as efficient thermal management of flexible electronics and soft robotics.

## Methods

### Synthesis of cubic BAs crystals

BAs crystals were prepared through chemical vapor transport. High-purity boron and arsenic coarse powders (Alfa Aesar) were ground by using mortar and pestle, prior to introduction into a quartz tube at a stoichiometric ratio of 1:2. After we loaded our reaction sources, the quartz tube was evacuated and flame sealed under high vacuum (10^−5^ Torr) before placement into a customized three-zone furnace for synthesis around 1033~1058 K. More details regarding the synthesis can be found in our recent publication^3^. The particle sizes of BAs crystals can be well controlled using growth conditions and, for this work the size range of BAs crystals is mainly distributed around 5~10 μm (inset, Fig. 2b).

### Fabrication of BAs polymetric composites

BAs aqueous slurry was prepared through the mixture of BAs powders and the solution, followed by sonication for 1 h and degasification in vacuum. Next, the BAs aqueous slurry was transferred into a sealed mold and frozen directionally using liquid nitrogen. Further, the sample was dehydrated using the freeze-drying process for 48 h in a freeze-dryer (Labconco, USA) to form the BAs assembly. The epoxy resin monomer (EPIKOTE Resin 862) was mixed with the curing agents (EPIKURE Curing Agent W) at a weight ratio of 100/20, and then infiltrated into the BAs assembly. After that, the sample was cured at 80 °C and 120 °C each for 2 h to form a composite structure of s-BAs. In addition, s-BAs samples were prepared and measured using different polymer matrixes, including epoxy, polydimethylsiloxane (PDMS), and elastomer (Ecoflex), and they show consistent thermal measurement results.

### Structural characterization of materials

SEM images were obtained with a field-emission SEM instrument (SU-3500, Hitachi). Transmission electron microscope (TEM) samples were prepared by using a focused ion beam (FIB) machine (Nova 600, FEI). After cleaning, we took the high-angle annular dark-field (HAADF) image by using aberration-corrected high-resolution scanning TEM (Grand ARM, JEOL, 300 kV).

### Thermal measurements

Specific heat is measured using a differential scanning calorimeter (TA Instruments, 2920) with a temperature increase rate of 5 °C/min from room temperature to 100 °C. Thermal diffusivity were measured using a standard laser flash setup, where a pulse laser irradiation was used to heat the composites from one side, and time-dependent temperature was recorded at the back end. For laser flash measurement, experimental conditions are carefully designed to ensure reliable analysis. Cross-validation on both thick and thin samples are performed and show consistent measurement results. For thick samples, a large laser-heating size and insulated sample boundary are applied, so that the whole sample is uniformly heated up and heat conduction is through the whole cross-section making a one-dimensional temperature profile. Meanwhile, the samples were placed in vacuum to avoid the heat loss to the environment. By recording the temperature rise at the rear side, the thermal diffusivity α can be calculated by the following Eq. (1):

$$\alpha = \frac{{1.38d^2}}{{\pi ^2t_{0.5}}}$$

where *d* is the sample thickness and *t*_0.5_ is the characteristic time for the sample to heat up to the half of the maximum temperature on its rear surface. The thermal conductivity (κ) can be determined after the measurement of mass density ρ, specific heat *c*_*p*_, and the thermal diffusivity α, following Eq. (2): κ = αρ*c*_*p*_.

### Thermal images of device temperatures

Transient temperature distributions near the hot spot of LED devices were taken by a calibrated infrared camera (FLIR A655sc). For comparison, three thermal interfaces including commercial thermal epoxy, silicone pad, and s-BAs were used. All samples were prepared in the same size and the LED chips were operating under the same conditions. The surface temperatures were calculated directly from the obtained videos and images by using the FLIR Tools+ (FLIR) and ImageJ (NIH) software packages. All variants of the experiments were performed for at least three different videos, verified with consistent results obtained in each instance.

### Thermal modeling

The effective thermal conductivity of the composite materials with varying extents of alignment is modeled by solving the heat conduction equation using the finite element method. The positions of BAs particles are distributed using random functions. The extent of alignment is quantified by the standard deviation of BAs particles to the averaged centerline upon alignment. Normal distribution function is used in the direction perpendicular to the alignment. The volume-averaged heat flux density over the whole domain was calculated under a given temperature gradient, and was consequently used to determine the effective thermal conductivity. The input thermal conductivity and specific heat used in this modeling are all measured from the experiment.

### Mechanical measurements

Mechanical properties were measured in the tensile mode with an Instron 5542 mechanical tester (Instron Corp., Norwood, MA) with a gauge length of 10 mm at a loading rate of 1 mm/min. All the samples were cut into 30 mm long segments. At least three samples were tested for each experimental condition to obtain statistically reliable values.

### Mechanical simulation

The Young’s modulus and shear modulus were modeled using the finite element method^36^, and under the same geometric model as the thermal model. For simulation, one end of the structure is fixed and the other end is applied with force to give the deformation. For the simulation of Young’s modulus, a normal force is applied and the axial deformation of the structure is computed. For simulation of the shear modulus, a shear force is applied and the shear deformation of the structure is computed. The effect of alignment of BAs particles on the mechanical properties were examined in the same setting as the thermal modeling, where the extent of alignment is quantified by the standard deviation of BAs particles.

## Supplementary information

Supplementary Information

## Data Availability

The authors declare that the main data supporting the findings of this study are contained within the paper and its associated Supplementary Information. All other relevant data are available from the corresponding author upon reasonable request.
