# Supplementary material for: Flexible thermal interface based on self-assembled boron arsenide for high-performance thermal management
Source: Nat Commun. 2021 Feb 24;12:1284. doi: 10.1038/s41467-021-21531-7 (PMC7904764; doi:10.1038/s41467-021-21531-7)
Supplement: Supplementary file 1 — Supplementary Information [file 41467_2021_21531_MOESM1_ESM.pdf]

Supplementary Information for

Flexible Thermal Interface Based on Self-Assembled Boron Arsenide for High-Performance Thermal Management

Ying Cui[1], Zihao Qin[1], Huan Wu[1], Man Li[1], Yongjie Hu[1]*

[1]Department of Mechanical and Aerospace Engineering
University of California, Los Angeles (UCLA), CA 90095, USA.

*Corresponding author. Email: yhu@seas.ucla.edu

Supplementary Figure

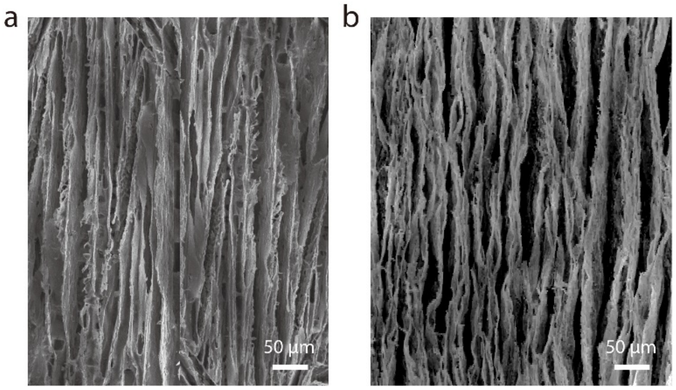

Figure S1. Cross-section SEM images of the s-BAs lamellar structures: (a) after 100 bending cycles; (b) after 500 bending cycles.

Supplementary Table

Table S1. Volume loading-dependent mass density, specific heat, thermal diffusivity, and thermal conductivity of the s-BAs samples.

BAs loading (vol %)	Mass density ρ (kg/m^3)	Specific heat c_p (J/kg·K)	Thermal diffusivity α (m^2/s)	Thermal conductivity κ (W/m·K)
5	1401	1141	1.357×10^{-6}	2.169
10	1602	1014	1.938×10^{-6}	3.148
15	1803	915.7	3.370×10^{-6}	5.565
20	2004	836.9	4.120×10^{-6}	6.910
30	2406	719.0	7.289×10^{-6}	12.61
40	2808	634.8	1.169×10^{-5}	20.83

Table S2. Thermal conductivity and anisotropy of s-BAs with different BAs loadings.

BAs loading (vol %)	Thermal conductivity along the aligned BAs crystals (W/m·K)	Thermal conductivity in perpendicular to aligned BAs crystals (W/m·K)
5	2.169	0.802
10	3.148	1.298
15	5.565	1.347
20	6.910	1.828
30	12.61	3.211
40	20.83	5.240

Finite element simulation of thermal conductivity

To quantify the effect on the thermal conductivity of s-BAs samples from varying particle alignment, we performed finite element modeling following several steps. First, simulation geometries with different extents of alignment in 3D space are generated by using random function to determine the position of each particles. The position of the particles follows normal distribution in radial direction, and uniform distribution in vertical direction. The extents of alignment are quantified by the standard deviation of the particle radial positions (see example schematics in Figure S2). For boundary conditions, the temperatures of the top and bottom surfaces are fixed and the volume-averaged heat flux density over the whole domain are calculated. To get reliable effective thermal conductivity, a large set of structures for each BAs loading and alignment degree are generated and a converged average value for the effective thermal conductivity is achieved to be within ~ 5%.

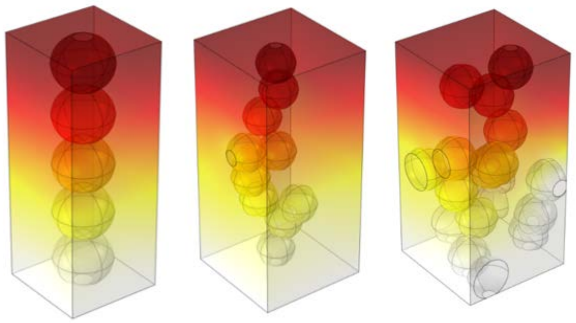

Figure S2. Schematics of example structures for the thermal conductivity simulation. From left to right, the extents of alignment decrease. The color indicates temperature distribution.
